# Case series of Li-Fraumeni syndrome: carcinogenic mechanisms in breast cancer with *TP53* pathogenic variant carriers

**DOI:** 10.1007/s12282-024-01612-3

**Published:** 2024-07-17

**Authors:** Mari Hosonaga, Eri Habano, Hiromi Arakawa, Keika Kaneko, Takeshi Nakajima, Naomi Hayashi, Ippei Fukada, Akira Nakamura, Yurie Haruyama, Tetsuyo Maeda, Hitoshi Inari, Takayuki Kobayashi, Eri Nakashima, Takayuki Ueno, Toshimi Takano, Shunji Takahashi, Shinji Ohno, Arisa Ueki

**Affiliations:** 1 Department of Breast Medical Oncology, Cancer Institute Hospital of Japanese Foundation for Cancer Research, Tokyo, 135-8550 Japan; 2 Department of Clinical Genetic Oncology, Cancer Institute Hospital of Japanese Foundation for Cancer Research, Tokyo, 135-8550 Japan; 3 Medical Ethics and Medical Genetics, Kyoto University, Graduate of School of Medicine, School of Public Health, Kyoto, 606-8501 Japan; 4 Department of Breast Surgery, Cancer Institute Hospital of Japanese Foundation for Cancer Research, Tokyo, 135-8550 Japan; 5 Division of Genomic Medicine, Cancer Institute Hospital of Japanese Foundation for Cancer Research, Tokyo, 135-8550 Japan

**Keywords:** Li-Fraumeni syndrome, *TP53*, Breast cancer, Hormone receptor, Human epidermal growth factor receptor 2

## Abstract

**Background:**

Li-Fraumeni syndrome (LFS), a hereditary condition attributed to *TP53* pathogenic variants,(PV), is associated with high risks for various malignant tumors, including breast cancer. Notably, individuals harboring TP53 PVs are more likely (67–83%) to develop HER2 + breast cancer than noncarriers (16–25%). In this retrospective study, we evaluated the associations between *TP53* variants and breast cancer phenotype.

**Methods:**

We conducted a retrospective review of the medical records of patients with LFS treated at a single institution and reviewed the literature on *TP53* functions and the mechanisms underlying HER2 + breast cancer development in LFS.

**Results:**

We analyzed data for 10 patients with LFS from 8 families. The median age at the onset of the first tumor was 35.5 years. Only case 2 met the classic criteria; this patient harbored a nonsense variant, whereas the other patients carried missense variants. We observed that 9 of 10 patients developed breast cancer. Immunohistochemical analyses revealed that 40% of breast cancers in patients with LFS were HR − /HER2 + . The median age at the onset of breast cancer was slightly younger in HR − /HER2 + tumors than in HR + /HER2 −  tumors (31 years and 35.5 years, respectively).

**Conclusions:**

The occurrence of HER2 + breast cancer subtype was 40% in our LFS case series, which is greater than that in the general population (16–25%). Some *TP53* PVs may facilitate HER2-derived oncogenesis in breast cancer. However, further studies with larger sample sizes are warranted to clarify the oncogenic mechanisms underlying each subtype of breast cancer in *TP53* PV carriers.

## Introduction

### *TP53* and Li-Fraumeni syndrome

Tumor protein P53 (*TP53)* expression is induced by various physiological stresses, such as DNA damage, radiation, hypoxia, and oncogene signaling. *TP53* functions as a guardian of the genome by inducing apoptosis and preventing the accumulation of genomic mutations in cells [1, 2]. Somatic pathogenic variants in *TP53* are found in 18–50% of all malignant tumors in humans [3, 4], suggesting that *TP53* is an early oncogenic driver in various types of cancers.

The p53 protein functions as a tumor suppressor primarily by binding to p53 DNA-binding sites in its target genes to regulate their expression [5]. *TP53* pathogenic variants (PVs), both germline and somatic, are primarily distributed in the DNA-binding domain and impair *TP53* transcription [6].

Li-Fraumeni syndrome (LFS) is a hereditary genetic condition attributed to *TP53* PVs. It is associated with high risks for a diverse spectrum of malignant tumors, including breast cancer. The frequency of *TP53* germline pathogenic variants in the general population is 0.03–0.27% [7–9].

### Role of TP53 in breast cancer development

Breast cancer is the most frequent type of cancer observed in patients with LFS, accounting for 79% of cancers among female *TP53* PV carriers [10].

In a study of human high-grade ductal carcinoma in situ (DCIS), a precursor lesion of invasive ductal carcinoma (IDC), the p53 pathway was inactivated in all DCIS specimens [11]. Breast cancer is assumed to originate from mammary epithelial cells [12]. Upon the depletion of mutant *TP53* in breast cancer cells, the irregular morphology, which is a hallmark of cancer, returns to a normal mammary epithelium-like structure. This implies that mutant *TP53* contributes to the disruption of the mammary tissue architecture during breast tumorigenesis [13].

Based on immunohistochemical (IHC) analyses of estrogen receptor (ER), progesterone receptor (PR), and human epidermal growth factor receptor 2 (HER2), breast cancer can be classified into four basic subtypes: hormone receptor (HR: ER or/and PR) + /HER2 − , HR − /HER2 + , HR + /HER2 + , and triple-negative (negative for ER, PR, and HER2), with frequencies of 68.9%, 7.5%, 10.2%, and 13.4%, respectively [14].

*TP53* PV carriers have a considerably higher rate of HER2 + tumors (67–83%) than that of noncarriers (16–25%) [15, 16]. However, the mechanism underlying HER2 overexpression in LFS-associated breast cancer remains unknown. Herein, we describe a case series of LFS in our institution and review the existing literature on *TP53* functions and the mechanism underlying HER2 + breast cancer development in LFS.

## Materials and methods

### Case series of LFS

We conducted a retrospective review of the medical records of patients with LFS diagnosed at our institution between July 2006 and August 2021. Among 11 *TP53* PV carriers, we analyzed 10 patients, excluding one unaffected carrier. We assessed the pathogenicity of *TP53* germline variants reported from commercial laboratories and verified with ACMG/AMP guidelines (Table 1) [17].

**Table 1:** Classification of *TP53* variants according to ACMG/AMP guidelines

	*TP53* variants (NM_000546.6)	ClinVar ID	ClinVar classifications	Germline variant classification by VarSome	Criteria applied (ACMG/AMP guidelines) [17]
1	c.638G > A (p.R213Q)	135,359	P	Pathogenic	PP5, PM1, PM5, PP3, PS3, PM2
2	c.1024C > T (p.R342*)	182,970	P	Pathogenic	PVS1, PP5, PM2
3	c.817C > T (p.R273C)	43,594	P/LP	Pathogenic	PP5, PM1, PM5, PP3, PS3, PM2
4	c.797G > A (p.G266E)	161,516	P/LP	Pathogenic	PP5, PM1, PM5, PP3
5	c.1009C > T (p.R337C)	142,536	P/LP	Pathogenic	PP5, PM5, PP3, PM1, PS3, PM2
6	c.743G > A (p.R248Q)	12,356	P	Pathogenic	PP5, PM1, PM5, PP3, PS3, PM2
7	c.473G > A (p.R158H)	141,963	P/LP	Pathogenic	PP5, PM1, PM5, PP3, PS3,PM2

### Clinical criteria

The classic LFS criteria were used for the diagnosis of LFS [18]. Despite carrying a germline *TP53* PV, several patients did not meet these criteria*.* Hence, the 2015 version of Chompret criteria [10] is widely used for identifying candidates for *TP53* germline genetic testing.

## Results

### Case series

We identified 10 *TP53* PV carriers with a history of cancer from eight families at our institution (Table 2). Cases 8–10 were from the same family, whereas the others were from different families. All patients were women, and the median age at the onset of the first tumor was 35.5 years (range: 8–45 years). Only case 2 met the classic criteria, whereas 50% of patients and 63% of families met the 2015 Chompret criteria. We found 25 tumors among the 10 patients with LFS, and the median number of tumors was two. The distribution of tumors was as follows: breast (15), bones (2), stomach (1), lung (2), colorectum (2), endometrial (1), ovary (1), and pancreas (1) (Table 2).

**Table 2:** Patient characteristics in the LFS case series

No	Sex	last follow-up age	Age (genetic testing)	1st tumor	2nd tumor	3rd tumor	4th and later	Classic criteria	Chompret criteria	TP53 variant (NM_000546.6)	Molecular consequence from IARC TP53 database	Age at brest cancer	Bilateral breast cancer	Breast cancer phenotype and treatment			Family history with breast cancer	
														Right	Left			
1	F	73	55	Chondrosarcoma (29 years)	Right breast cancer (39 years)	Left breast cancer (44 years)	*Endometrial cancer (48 years) etc	No	Yes	c.638G > A (p.R213Q)	Misssense	39	Yes	IDC, unknown for ER/PR/HER2	DCIS		No	N/A
														Bilateral Bt + Ax, RT (−)				
2	F	38	29	Osteosarcoma (8 years)	Left breast cancer (28 years)	Lung cancer (34 years)	Left breast cancer (37 years)	Yes	Yes	c.1024C > T (p.R342*)	Nonssense	28	No	N/A	IDC, ER − /PR − /HER2:3 +	IDC, ER + /PR + / HER2:0	Yes	Mother at 60’s, and paternal grandmother at 30’s
															Bp + SNB, RT (−)	Bt + SNB, RT (−)		
3	F	50	40	Bilateral breast cancer (34 years)	Lung cancer (37 years)	N/A	N/A	No	No	c.817C > T (p.R273C)	Misssense	34	Yes	DCIS	IDC, ER − /PR − /HER2:3 +		No	N/A
														Bilateral Bt + SNB, RT (−)				
4	F	36	26	Left breast cancer (26 years)	Right breast cancer (34 years)	N/A	N/A	No	Yes	c.797G > A (p.G266E)	Misssense	26	No	IDC, ER + /PR + /HER2: 1 +	IDC, ER + /PR + /HER2: 0		NO	N/A
														Bt + SNB, RT (−)	Bt + Ax, RT ( +)			
5	F	47	41	Left breast cancer (41 years)	Colon cancer (47 years)	N/A	N/A	No	Yes	c.1009C > T (p.R337C)	Misssense	41	No	N/A	IDC, ER − /PR − /HER2: 3 +		Yes	Bilateral breast cancer at 50’s in mother
															Bt + SNB, RT (−)			
6	F	37	35	Left breast cancer (28 years)	Right breast cancer (34 years)	N/A	N/A	No	Yes	c.743G > A (p.R248Q)	Misssense	28	Yes	DCIS	IDC, ER − /PR − /HER2: 3 +		No	N/A
														Bt + SNB, RT (−)	Bt + Ax, RT ( +)			
7	F	58	56	Ovarian cancer (53 years)	Pancreatic cancer (54 years)	N/A	N/A	No	No	c.743G > A (p.R248Q)	Misssense	N/A	N/A	N/A	N/A		Yes	Mother at 60’s
8	F	38	34	Left breast cancer (33 years)	N/A	N/A	N/A	No	No	c.473G > A (p.R158H)	Misssense	33	No	N/A	IDC, ER + /PR + /HER2: 0		Yes	Mother at 30’s, and maternal grandmother at 40’s
															Bt + SNB, RT (−)			
9	F	47	43	Bilateral breast cancer (43 years)	N/A	N/A	N/A	No	No			43	Yes	DCIS	ILC, ER + /PR + /HER2: 1 +			
														Bilateral Bt + SNB, RT (−)				
10	F	40	37	Left breast cancer (35 years)	N/A	N/A	N/A	No	No			35	No	N/A	IDC, ER + /PR + , HER2: 2 + , FISH −			
															Bp + Ax, RT (−)			

*Case 1 also had endometrial cancer at the age of 48 years, gastric cancer at the age of 55 years, and colorectal cancer at the age of 71 years.

Case 7 was diagnosed with LFS after tumor genomic profiling. She had ovarian cancer at the age of 53 years and pancreatic cancer at the age of 54 years. The patient received somatic tumor panel testing at the age of 54 years, which indicated potential germline *TP53* PVs. Thereafter, genetic testing confirmed that she carried a c.743G > A (p.R248Q) variant, the same variant detected in case 6.

### Breast *cancer* characteristics

We observed 15 breast cancers in 9 out of 10 *TP53* PV carriers (Table 2). The median age at the onset of breast cancer was 34 years (range: 26–45 years). The pathological features of the 15 breast cancers were as follows: IDC (10), invasive lobular carcinoma (ILC) (1), and DCIS (4). According to IHC analyses of 11 invasive breast cancer specimens, excluding 1 tumor that was not assessed by IHC (Case 1), 40% (4 out of 10) of tumors were HR − /HER2 + , whereas the remaining 60% were the HR + /HER2- subtype. Furthermore, among all patients with breast cancer, 56% (five of nine) had bilateral breast cancer and 67% (six of nine) had a family history of breast cancer in their mothers (Table 2).

**Fig. 1:**
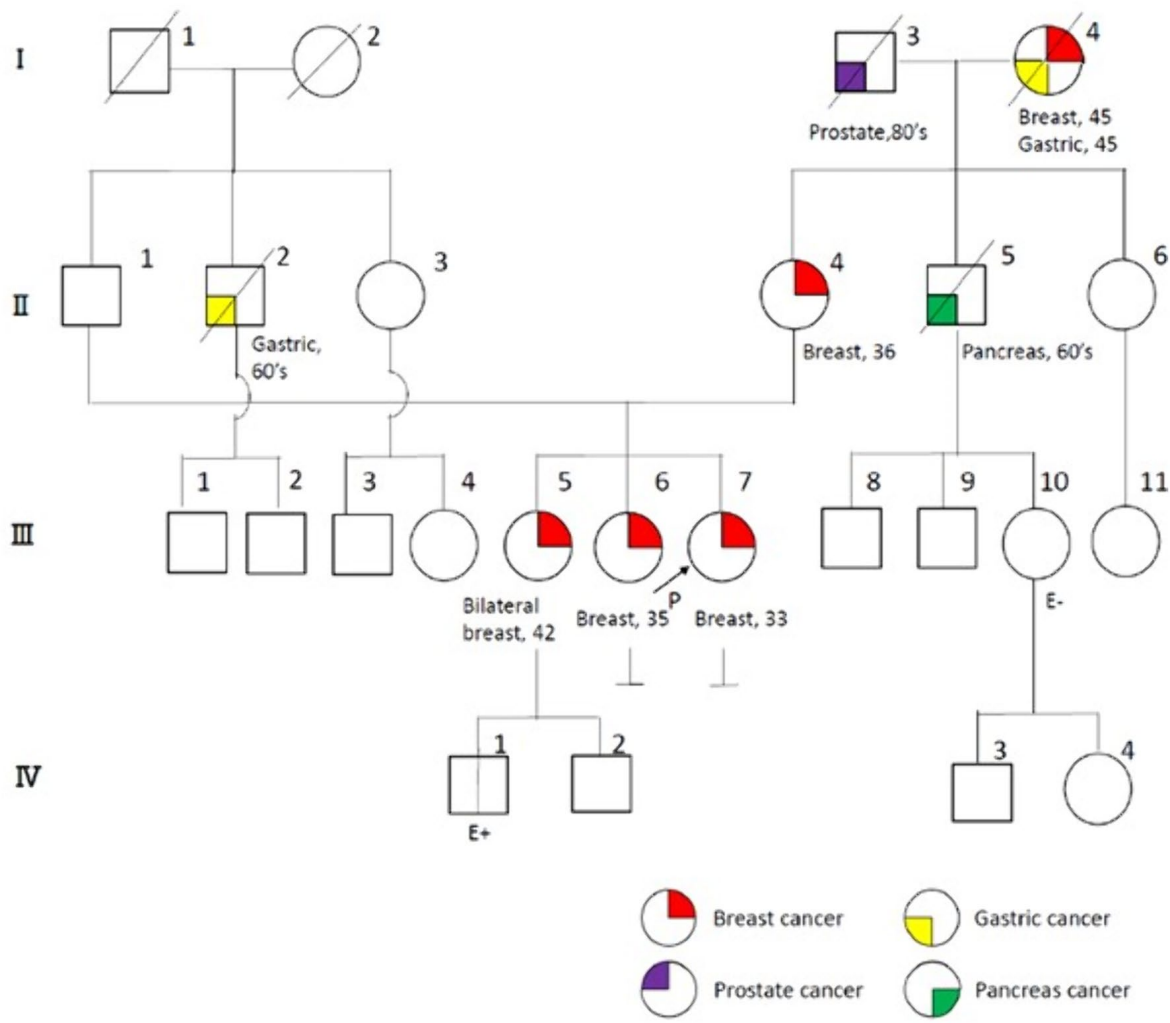
Family tree of cases 8–10. According to the family history of case 8, the mother was diagnosed with breast cancer at 36 years, maternal uncle with pancreatic cancer at 60 years, and maternal grandmother with breast and gastric cancers at 45 years of age. IV-1 was an unaffected carrier with the same pathogenic *TP53* variant.

Case 8, diagnosed with ER + /PR + /HER2- breast cancer at 33 years, was a proband in this family carrying a c.473G > A (p.R158H) variant. The elder sisters of the patient (cases 9 and 10), who later underwent a *TP53* analysis, also carried the same *TP53* PV. In case 9, the first surveillance breast MRI detected bilateral breast cancer at the age of 43 years; DCIS in the right breast and ER + /PR + /HER2 −  ILC in the left breast. Case 10 had a history of ER + /PR + /HER2 −  IDC diagnosed at the age of 35 years (Table 2, Fig. 1).

### Radiotherapy for breast *cancer*

Among 15 breast cancers, only 2 tumors were treated with radiotherapy (RT). Case 4 had bilateral breast cancer. The first tumor was left breast cancer at 26 years (Stage III, IDC, ER + /PR + /HER2 − ) treated with neoadjuvant chemotherapy with anthracycline followed by taxane, then by mastectomy with axillary lymph node dissection (Ax), with residual cancer in five nodes. Adjuvant RT and endocrine therapy were administered.

Case 6 also had bilateral breast cancer, the first tumor was left breast cancer (Stage III, IDC, ER − /PR − /HER2 +) at 28 years treated with neoadjuvant chemotherapy with anthracycline followed by a taxane plus trastuzumab regimen, and then mastectomy with Ax, with no invasive residual cancer in the breast or lymph nodes. Adjuvant trastuzumab and RT were administered.

In case 2, left breast cancer was diagnosed at 27 years and treated with breast-conserving surgery and sentinel node biopsy (no metastasis), pathological findings revealed ER − /PR − /HER2 + IDC. She did not undergo RT because we identified that she had a *TP53* c.1024C > T (p.R342*) variant after surgery. A new primary breast cancer, ER + /PR + /HER2 −  IDC, was detected in her remaining left breast at 38 years.

## Discussion

### Tumor distribution in *TP53* pathogenic variant carriers

In our study, 63% of families with LFS met the 2015 Chompret criteria (Table 2) with a lower positivity rate than that previously reported; the sensitivity of the 2009 Chompret criteria is 57–82% [10, 19]. In the largest investigation of LFS in Japan (68 individuals from 48 families), 60.4% of families met the 2015 Chompret criteria [20], comparable with our results. They reported lower frequencies of soft tissue sarcoma (7.8% vs. 19.0%) and breast cancer (19.5% vs. 31.4%) in Japanese patients than in French patients with LFS [20]. In our study, 90% of patients with LFS were affected by breast cancer, accounting for 60% of all tumors (15 out of 25). Notably, these data may be biased because our institution specifically treats patients with cancer, and the number of patients with breast cancer is particularly high. This may explain why all the patients were women in this study as well as the high probability of breast cancer.

### *TP53* hot spots and the distribution of TP53 variants

**Fig. 2:**
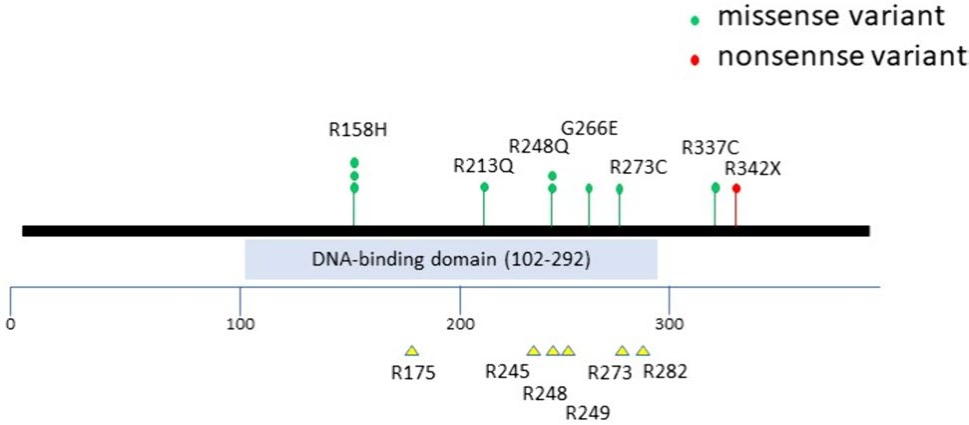
*TP53* hotspots. Six previously reported *TP53* hotspots in human cancers from the IARC database are described in the lower part [6]. The spectrum of *TP53* variants in our case series is shown in the upper part: green circles indicate missense variants, whereas red circles indicate nonsense variants

The distribution of *TP53* PVs is shown in Fig. 2. We found that 3 out of 10 patients carried variants in sites previously reported as mutation hotspots, such as R175, R245, R248, R249, R273, and R282, corresponding to the p53 DNA-binding domain [6]. Most (75%) *TP53* somatic variants are missense variants [5]. In our study, 9 out of 10 patients carried a missense variant, whereas one patient carried a nonsense variant (Fig. 2).

In our study, only case 2 had a nonsense variant; this was the only case involving a history of sarcoma and meeting the classic LFS criteria in our case series (Table 2; Fig. 2). These findings were consistent with the results obtained by Rana et al., who reported that loss-of-function variants were associated with an earlier tumor onset, increased frequency of sarcoma, and higher rate of meeting classic LFS criteria than missense variants [21].

### *TP53* hot spot variants facilitate HER2-derived oncogenesis

In vivo, *Trp53 PV* knock-in mice (R175H, R273H, and R248Q) exhibit a higher tumor bulk with an increased grade and invasion, metastatic ability, and shorter life span than those of *Trp53*-null mice [22, 23]. These *Trp53* PVs have also been identified in humans [6] (Fig. 2).

HER2 overexpression in breast cancer activates pathways that promote cell proliferation, reduce apoptosis, and increase metastasis [24]. In vitro, p53 variants (R248Q and R273C) increase *HER2* expression, whereas the suppression of PV *TP53* reduces *HER2* expression and inhibits the downstream pathway [25]. In a HER2 transgenic mouse model, a PV *Trp53* allele induces the formation of multicentric mammary tumors and leads to early tumor onset and short survival [26].

Notably, somatic *TP53* PVs are most frequent in breast cancer, reaching incidence rates of 28–37% [27, 28], and are especially frequent in the HER2 + than in the HR + subtype (72%, and 12–29%, respectively) [27]. An analysis of the HER2 + breast cancer dataset revealed that the level of *HER2* mRNA expression is also considerably higher in tumors expressing *TP53* PV than in tumors expressing the wild-type *TP53* [29]. In our study, all HER2 + cases (4 out of 4 tumors) were graded as IHC 3 + , indicating high *HER2* mRNA expression. Most HER2 IHC 3 + cases (94.7%) have been reported to show HER2 amplification by FISH [30].

These previous reports suggest that specific *TP53* PVs facilitate HER2-derived oncogenesis and cancer progression in HER2 + breast cancer, potentially resulting in a high proportion of HER2 + breast cancers in patients with LFS.

### Mechanisms underlying both HER2 + and HR + tumors arising in TP53 PV carriers

Although there were no HR + /HER2 + tumors in our study, HR + /HER2 + tumors are also more frequent in patients with LFS than in the general population (53% and 10%, respectively) [31, 32]. In vitro, ER binds to wild-type TP53 directly and represses its transcriptional activation [33]. Conversely, estrogen increases p53 protein levels, whereas estrogen deprivation reduces p53 levels [34]. Thus, contradictory results have been obtained regarding the relationship between ER and wild-type *TP53.*

Regarding the relationship between ER and TP53 PV, limited data exist; estrogen increases TP53 *PV* protein expression, whereas estrogen deprivation reduces TP53 expression levels [34]. In *TP53* PV carriers, estrogen-ER signaling might affect the early onset of breast cancer; both ER and HER2 signaling are drivers of cell proliferation and disease progression in breast cancer, and their crosstalk might facilitate breast cancer development; however, further investigations are required to clarify these relationships.

### Age at breast *cancer* onset in TP53 PV carriers

The median age of *TP53* PV carriers at the time of diagnosis of breast cancer appears to be similar in Japan and France: the median ages were 34 years in our study, 32 years in the study by Funato et al. [20], and 33 years in a study of the French LFS working group [10]. The median age in all three independent studies was over 31 years, which is the age criterion considered for the *TP53* genetic test according to the Chompret criteria.

The reported prevalence of *TP53* PVs is 2.2–4.0% in women with breast cancer before the age of 31 years [35, 36]. In a study focused on HER2 + breast cancer, the prevalence of *TP53* PV is 3% in patients diagnosed before 41 years; however, the prevalence increases to 8.5% in patients diagnosed before the age of 31 years [37]. In our study, the median age at the onset of breast cancer was slightly younger for HR − /HER2 + tumors than for HR + /HER2 −  tumors (31 years and 35.5 years, respectively), although our study was limited by a small sample size. This suggests that *TP53* genetic testing should be considered for patients with breast cancer at a slightly older age than 31 years, especially in HER2 + breast cancer. Similarly, Evans et al. suggested new criteria for *TP53* germline testing to include women diagnosed with HER2 + breast cancer before the age of 36 years [38]. At our institution, *TP53* germline testing is recommended for women diagnosed with breast cancer who are negative for BRCA1 and BRCA2, regardless of subtype, before 31 years of age.

### Radiotherapy

*TP53* PV carriers are susceptible to a high risk of radiation-induced secondary malignancy after RT. The LFS guideline recommends avoiding radiotherapy when possible [39]. In our study, two cases (cases 4 and 6) underwent adjuvant RT due to axillary lymph node metastasis and the high risk of recurrence. The patients did not have any tumors within the radiation field at follow-up times of 10 years and 9 years after RT. However, longer follow-up periods are needed because the period of radiation-induced secondary malignancy is 3–22 years (median 7 years) [40].

Mastectomy, rather than lumpectomy, is preferable to avoid a second malignancy; however, for a *TP53* PV carrier with advanced breast cancer, adjuvant RT should be considered carefully depending on the risk of recurrence.

## Conclusions

In summary, our study included a relatively large LFS case series from a single institution. The HER2 + breast cancer subtype was more frequent in patients with LFS (40%) than in patients with sporadic breast cancer (16–25%), consistent with previous studies. HER2 signaling is a well-known driver of cell proliferation and progression in breast cancer. Previous reports suggest that *TP53* PVs facilitate HER2-derived oncogenesis, which might account for the high proportion of HER2 + breast cancer in *TP53* PV carriers. It also might explain the slight difference in the onset of breast cancer between HR − /HER2 + and HR + /HER2 −  tumors in our study. A limitation of our study was the small sample size at a single institution, and future investigations are warranted to clarify the oncogenic mechanisms in each subtype of breast cancer. Considering the rarity of germline *TP53* PVs, clinical data collection at multiple institutions in several countries as an international collaborative study is desirable.

## Data Availability

Data available on request due to privacy restrictions. The variant information that support the findings of this study are openly available in Table1 and 2. Some data that support the findings of this study are available on request from the corresponding author, MH. The data are not publicly available due to restrictions their containing information that could compromise the privacy of research participants.

## References

[1] Takaoka A, Hayakawa S, Yanai H, Stoiber D, Negishi H, Kikuchi H, et al. Integration of interferon-alpha/beta signalling to p53 responses in tumour suppression and antiviral defence. Nature. 2003;424:516–23. 10.1038/nature01850. 12872134 10.1038/nature01850

[2] Karimian A, Ahmadi Y, Yousefi B. Multiple functions of p21 in cell cycle, apoptosis and transcriptional regulation after DNA damage. DNA Repair. 2016;42:63–71. 10.1016/j.dnarep.2016.04.008. 27156098 10.1016/j.dnarep.2016.04.008

[3] Levine AJ, Oren M. The first 30 years of p53: growing ever more complex. Nat Rev Cancer. 2009;9:749–58. 10.1038/nrc2723. 19776744 10.1038/nrc2723 PMC2771725

[4] Mandelker D, Donoghue M, Talukdar S, Bandlamudi C, Srinivasan P, Vivek M, et al. Germline-focussed analysis of tumour-only sequencing: recommendations from the ESMO Precision Medicine Working Group. Ann Oncol. 2019;30:1221–31. 10.1093/annonc/mdz136. 31050713 10.1093/annonc/mdz136 PMC6683854

[5] Yue X, Zhao Y, Xu Y, Zheng M, Feng Z, Hu W. Mutant p53 in cancer: accumulation, gain-of-function, and therapy. J Mol Biol. 2017;429:1595–606. 10.1016/j.jmb.2017.03.030. 28390900 10.1016/j.jmb.2017.03.030 PMC5663274

[6] Freed-Pastor WA, Prives C. Mutant p53: one name, many proteins. Genes Dev. 2012;26:1268–86. 10.1101/gad.190678.112. 22713868 10.1101/gad.190678.112 PMC3387655

[7] de Andrade KC, Mirabello L, Stewart DR, Karlins E, Koster R, Wang M, et al. Higher-than-expected population prevalence of potentially pathogenic germline TP53 variants in individuals unselected for cancer history. Hum Mutat. 2017;38:1723–30. 10.1002/humu.23320. 28861920 10.1002/humu.23320 PMC6858060

[8] Yamaguchi-Kabata Y, Yasuda J, Tanabe O, Suzuki Y, Kawame H, Fuse N, et al. Evaluation of reported pathogenic variants and their frequencies in a Japanese population based on a whole-genome reference panel of 2049 individuals. J Hum Genet. 2018;63:213–30. 10.1038/s10038-017-0347-1. 29192238 10.1038/s10038-017-0347-1

[9] Momozawa Y, Iwasaki Y, Parsons MT, Kamatani Y, Takahashi A, Tamura C, et al. Germline pathogenic variants of 11 breast cancer genes in 7051 Japanese patients and 11,241 controls. Nat Commun. 2018;9:4083. 10.1038/s41467-018-06581-8. 30287823 10.1038/s41467-018-06581-8 PMC6172276

[10] Bougeard G, Renaux-Petel M, Flaman JM, Charbonnier C, Fermey P, Belotti M, et al. Revisiting Li-Fraumeni syndrome from TP53 mutation carriers. J Clin Oncol. 2015;33:2345–52. 10.1200/JCO.2014.59.5728. 26014290 10.1200/JCO.2014.59.5728

[11] Abba MC, Gong T, Lu Y, Lee J, Zhong Y, Lacunza E, et al. A molecular portrait of high-grade ductal carcinoma in situ. Cancer Res. 2015;75:3980–90. 10.1158/0008-5472.CAN-15-0506. 26249178 10.1158/0008-5472.CAN-15-0506 PMC4768486

[12] Bissell MJ, Radisky DC, Rizki A, Weaver VM, Petersen OW. The organizing principle: microenvironmental influences in the normal and malignant breast. Differentiation. 2002;70:537–46. 10.1046/j.1432-0436.2002.700907.x. 12492495 10.1046/j.1432-0436.2002.700907.x PMC2933198

[13] Freed-Pastor WA, Mizuno H, Zhao X, Langerød A, Moon SH, Rodriguez-Barrueco R, et al. Mutant p53 disrupts mammary tissue architecture via the mevalonate pathway. Cell. 2012;148:244–58. 10.1016/j.cell.2011.12.017. 22265415 10.1016/j.cell.2011.12.017 PMC3511889

[14] Onitilo AA, Engel JM, Greenlee RT, Mukesh BN. Breast cancer subtypes based on ER/PR and Her2 expression: comparison of clinicopathologic featues and survival. Clin Med Res. 2009;7:4–13. 10.3121/cmr.2009.825. 19574486 10.3121/cmr.2009.825 PMC2705275

[15] Wilson JR, Bateman AC, Hanson H, An Q, Evans G, Rahman N, et al. A novel HER2-positive breast cancer phenotype arising from germline TP53 mutations. J Med Genet. 2010;47:771–4. 10.1136/jmg.2010.078113. 20805372 10.1136/jmg.2010.078113

[16] Melhem-Bertrandt A, Bojadzieva J, Ready KJ, Obeid E, Liu DD, Gutierrez-Barrera AM, et al. Early onset HER2-positive breast cancer is associated with germline TP53 mutations. Cancer. 2012;118:908–13. 10.1002/cncr.26377. 21761402 10.1002/cncr.26377 PMC3527897

[17] Richards S, Aziz N, Bale S, Bick D, Das S, Gastier-Foster J, et al. Standards and guidelines for the interpretation of sequence variants: a joint consensus recommendation of the American College of Medical Genetics and Genomics and the Association for Molecular Pathology. Genet Med. 2015;17:405–24. 10.1038/gim.2015.30. 25741868 10.1038/gim.2015.30 PMC4544753

[18] Li FP, Fraumeni JF Jr, Mulvihill JJ, Blattner WA, Dreyfus MG, Tucker MA, et al. A cancer family syndrome in twenty-four kindreds. Cancer Res. 1988;48:5358–62. 3409256

[19] Gonzalez KD, Noltner KA, Buzin CH, Gu D, Wen-Fong CY, Nguyen VQ, et al. Beyond Li Fraumeni syndrome: clinical characteristics of families with p53 germline mutations. J Clin Oncol. 2009;27:1250–6. 10.1200/JCO.2008.16.6959. 19204208 10.1200/JCO.2008.16.6959

[20] Funato M, Tsunematsu Y, Yamazaki F, Tamura C, Kumamoto T, Takagi M, et al. Characteristics of Li-Fraumeni syndrome in Japan; a review study by the special committee of JSHT. Cancer Sci. 2021;112:2821–34. 10.1111/cas.14919. 33932062 10.1111/cas.14919 PMC8253286

[21] Rana HQ, Clifford J, Hoang L, LaDuca H, Black MH, Li S, et al. Genotype-phenotype associations among panel-based TP53+ subjects. Genet Med. 2019;21:2478–84. 10.1038/s41436-019-0541-y. 31105275 10.1038/s41436-019-0541-y

[22] Lang GA, Iwakuma T, Suh YA, Liu G, Rao VA, Parant JM, et al. Gain of function of a p53 hot spot mutation in a mouse model of Li-Fraumeni syndrome. Cell. 2004;119:861–72. 10.1016/j.cell.2004.11.006. 15607981 10.1016/j.cell.2004.11.006

[23] Hanel W, Marchenko N, Xu S, Yu SX, Weng W, Moll U. Two hot spot mutant p53 mouse models display differential gain of function in tumorigenesis. Cell Death Differ. 2013;20:898–909. 10.1038/cdd.2013.17. 23538418 10.1038/cdd.2013.17 PMC3679454

[24] Sachdev JC, Jahanzeb M. Blockade of the HER family of receptors in the treatment of HER2-positive metastatic breast cancer. Clin Breast Cancer. 2012;12:19–29. 10.1016/j.clbc.2011.07.001. 21903480 10.1016/j.clbc.2011.07.001

[25] Román-Rosales AA, García-Villa E, Herrera LA, Gariglio P, Díaz-Chávez J. Mutant p53 gain of function induces HER2 over-expression in cancer cells. BMC Cancer. 2018;18:709. 10.1186/s12885-018-4613-1. 29970031 10.1186/s12885-018-4613-1 PMC6029411

[26] Yallowitz AR, Li D, Lobko A, Mott D, Nemajerova A, Marchenko N. Mutant p53 amplifies epidermal growth factor receptor family signaling to promote mammary tumorigenesis. Mol Cancer Res. 2015;13:743–54. 10.1158/1541-7786.MCR-14-0360. 25573952 10.1158/1541-7786.MCR-14-0360 PMC4824060

[27] Network CGA. Comprehensive molecular portraits of human breast tumours. Nature. 2012;490:61–70. 10.1038/nature11412. 23000897 10.1038/nature11412 PMC3465532

[28] Nik-Zainal S, Davies H, Staaf J, Ramakrishna M, Glodzik D, Zou X, et al. Landscape of somatic mutations in 560 breast cancer whole-genome sequences. Nature. 2016;534:47–54. 10.1038/nature17676. 27135926 10.1038/nature17676 PMC4910866

[29] Fedorova O, Daks A, Shuvalov O, Kizenko A, Petukhov A, Gnennaya Y, et al. Attenuation of p53 mutant as an approach for treatment Her2-positive cancer. Cell Death Discov. 2020;6:100. 10.1038/s41420-020-00337-4. 33083021 10.1038/s41420-020-00337-4 PMC7548004

[30] Gown AM, Goldstein LC, Barry TS, Kussick SJ, Kandalaft PL, Kim PM, et al. High concordance between immunohistochemistry and fluorescence in situ hybridization testing for HER2 status in breast cancer requires a normalized IHC scoring system. Mod Pathol. 2008;21:1271–7. 10.1038/modpathol.2008.83. 18487992 10.1038/modpathol.2008.83

[31] Lund MJ, Butler EN, Hair BY, Ward KC, Andrews JH, Oprea-Ilies G, et al. Age/race differences in HER2 testing and in incidence rates for breast cancer triple subtypes: a population-based study and first report. Cancer. 2010;116:2549–59. 10.1002/cncr.25016. 20336785 10.1002/cncr.25016

[32] Masciari S, Dillo DA, Rath M, Robson M, Weitzel JN, Balmana J, et al. Breast cancer phenotype in women with TP53 germline mutations: a Li-Fraumeni syndrome consortium effort. Breast Cancer Res Treat. 2012;133:1125–30. 10.1007/s10549-012-1993-9. 22392042 10.1007/s10549-012-1993-9 PMC3709568

[33] Sayeed A, Konduri SD, Liu W, Bansal S, Li F, Das GM. Estrogen receptor alpha inhibits p53-mediated transcriptional repression: implications for the regulation of apoptosis. Cancer Res. 2007;67:7746–55. 10.1158/0008-5472.CAN-06-3724. 17699779 10.1158/0008-5472.CAN-06-3724

[34] Fernandez-Cuesta L, Anaganti S, Hainaut P, Olivier M. Estrogen levels act as rheostat on p53 levels and modulate p53-dependent responses in breast cancer cell lines. Breast Cancer Res Treat. 2011;125:35–42. 10.1007/s10549-010-0819-x. 20221692 10.1007/s10549-010-0819-x

[35] Mouchawar J, Korch C, Byers T, Pitts TM, Li E, McCredie MRE, et al. Population-based estimate of the contribution of TP53 mutations to subgroups of early-onset breast cancer. Australian Breast Cancer Family Study. Cancer Res. 2010;70:4795–800. 10.1158/0008-5472.CAN-09-0851. 20501846 10.1158/0008-5472.CAN-09-0851 PMC3228832

[36] Bakhuizen JJ, Hogervorst FB, Velthuizen ME, Ruijs MW, van Engelen K, van Os TA, et al. TP53 germline mutation testing in early-onset breast cancer: findings from a nationwide cohort. Fam Cancer. 2019;18:273–80. 10.1007/s10689-018-00118-0. 30607672 10.1007/s10689-018-00118-0

[37] Eccles DM, Li N, Handwerker R, Maishman T, Copson ER, Durcan LT, et al. Genetic testing in a cohort of young patients with HER2-amplified breast cancer. Ann Oncol Off J Eur Soc Med Oncol. 2016;27:467–73. 10.1093/annonc/mdv592. 10.1093/annonc/mdv592 26681682

[38] Evans DG, Woodward ER, Bajalica-Lagercratz S, Oliveira C, Frebbourg T. Germline TP53 testing in breast cancers: why, when and how? Cancers. 2020;12:E3762. 10.3390/cancers12123762. 10.3390/cancers12123762 PMC7764913 33327514

[39] Frebourg T, Bajalica Lagercrantz S, Oliveira C, Magenheim R, Gareth Evans D, European Reference Network GENTURIS. Guidelines for the Li-Fraumeni and heritable TP53-related cancer syndromes. Eur J Hum Genet EJHG. 2020;28:1379–86. 10.1038/s41431-020-0638-4. 32457520 10.1038/s41431-020-0638-4 PMC7609280

[40] Hisada M, Garber TE, Fung CY, Fraumeni JF, Li FP. Multiple primary cancers in families with Li-Fraumeni syndrome. J Natl Cancer Inst. 1998;90:606–11. 10.1093/jnci/90.8.606. 9554443 10.1093/jnci/90.8.606

